# Physical activity in the prevention of human diseases: role of epigenetic modifications

**DOI:** 10.1186/s12864-017-4193-5

**Published:** 2017-11-14

**Authors:** Elisa Grazioli, Ivan Dimauro, Neri Mercatelli, Guan Wang, Yannis Pitsiladis, Luigi Di Luigi, Daniela Caporossi

**Affiliations:** 1 Department of Movement, Human and Health Sciences, Unit of Biology, Genetics and Biochemistry, University of Rome “Foro Italico”, Rome, Italy; 2 FIMS Reference Collaborating Centre of Sports Medicine for Anti-Doping Research, University of Brighton, Brighton, UK; 3 Department of Movement, Human and Health Sciences, Unit of Endocrinology, University of Rome “Foro Italico”, Rome, Italy

**Keywords:** DNA methylation, Histone modification, Exercise, Disease prevention

## Abstract

Epigenetic modification refers to heritable changes in gene function that cannot be explained by alterations in the DNA sequence. The current literature clearly demonstrates that the epigenetic response is highly dynamic and influenced by different biological and environmental factors such as aging, nutrient availability and physical exercise. As such, it is well accepted that physical activity and exercise can modulate gene expression through epigenetic alternations although the type and duration of exercise eliciting specific epigenetic effects that can result in health benefits and prevent chronic diseases remains to be determined. This review highlights the most significant findings from epigenetic studies involving physical activity/exercise interventions known to benefit chronic diseases such as metabolic syndrome, diabetes, cancer, cardiovascular and neurodegenerative diseases.

## Background

A useful definition of epigenetics is “the study of mitotically and/or meiotically heritable changes in gene function that cannot be explained by changes in DNA sequence” [1]. Although the standard definition of “epigenome” refers to the combination of chemical changes to DNA and histone proteins in a cell, epigenetic changes generally include functional modification of the genome driven by DNA methylation, histone modification and microRNA expression. Epigenetic changes represent flexible genomic parameters that can modify genome function and also provide a mechanism that allows for the stable propagation of gene activity status from one generation of cells to the next [2]. Initially, epigenomic processes were considered unidirectional, but recent studies have demonstrated that the epigenome is highly dynamic and changes in response to biological factors such as development and aging processes or under the influence of exogenous factors such as nutrient availability and physical exercise [3, 4]. Many studies have been performed in the last two decades to better understand this epigenetic modulation, and results to date suggest that this phenomenon is intricately linked to cellular processes such as DNA repair, differentiation and stress events, as well as the progression and treatment of many chronic and degenerative diseases including cancer [1, 5, 6].

The aims of this review is to analyse specifically the most significant findings in human beings highlighting the role of epigenetic mechanisms in the beneficial effects of physical activity (PA) towards the prevention or therapy of diseases such as cancer, metabolic, cardiovascular and neurodegenerative diseases. A detailed examination of the molecular pathways involved in epigenetic modifications, as well as of the general effect of PA on epigenetic modifications, overcomes the scope of this review and can be found elsewhere [7, 8].

## Overview of epigenetic changes

### DNA methylation

DNA methylation is the most studied epigenetic process that is responsible for the addition of a methyl group to the 5-carbon position of a cytosine base catalysed by a family of DNA methyltransferases. The bases highly susceptible to methylation are typically found within the Cytosine-phosphate-Guanine (CpG) dinucleotide sequence of DNA; the so-called CpG island. In human somatic cells, for example, 5-methyl-cytosine (m^5^C) accounts for 70–80% of all CpG dinucleotides in the genome [9]. This type of modulation alters the expression of genes in the cells, working as an “on-off switch”: when a specific CpG reach site (CpG island) is methylated the gene expression is silenced, conversely its demethylation allows gene expression [10]. In animals and humans, both the methylation levels and their specific pattern are very dynamic during different stages of life, thus influencing development and maturation through orchestrated events in combination with environmental input [11]. It is clear that the methylation process can play a key role in several biological processes, including X-chromosome inactivation, parental imprinting, development, silencing of foreign DNA, and proper chromosome segregation [5, 12]. Aberrant methylation patterns are for instance associated with many forms of abnormal growth of tissue via hypermethylation of promoters repressing the transcription of tumour suppressor genes [13], as well as by hypomethylation of retrotransposons leading to their activation and translocation in other genomic regions inducing chromosomal instability [14].

### Histone modification

Histones are high alkaline proteins composed of many amino acids with basic side chains (particularly Lysine and Arginine). Their tasks are packaging and ordering of DNA into structural units called nucleosomes and represent the chief protein components of chromatin. Histone modifications are post-translational alterations that include the acetylation or methylation of specific histone regions, especially H3 and H4 histones. In general, these alterations are reversible and associated with transcriptional activation [15, 16]. In particular, the lysine residues within the N-terminal tail protruding from the nucleosome core are acetylated and deacetylated as part of gene regulation. These reactions are typically catalysed either by histone acetyltranferase (HAT) or by histone deacetylase (HDAC) enzymes [5, 12]. Specifically, acetylation removes the positive charge of histones decreasing their interaction with the negatively charged phosphate group of DNA, leading to a more relaxed chromatin structure favouring enhanced gene transcription. This status can be reversed by HDAC able to promote the deacetylation process, which is associated with reduction of transcriptional activity [17]. Although there is little evidence dealing with histone methylation, several enzymes such as histone methyltransferases (HMTs), peptidylarginine deiminase 4 (PADI4), lysine-specific demethylase 1 (LSD1) and Jumonji C-domain-containing histone demethylase (JHDM) are known to add or remove methyl groups to lysine tail regions of histones [16].

### MicroRNA expression

MicroRNAs (miRNAs) are small RNA molecules, about 22 nucleotides long, able to negatively affect gene expression at the post-transcriptional level (or “in a post-transcriptional manner”). MicroRNAs repress the expression of a large number of messenger RNAs (mRNAs) by direct binding to specific sequences localised in the 3′ untranslated regions of these target mRNAs [18]. Each miRNA is predicted to have many targets, and each mRNA may be regulated by more than one miRNA. Depending on their expression profile, miRNAs contribute to several fundamental physiological and pathophysiological functions, including the regulation of developmental timing and pattern formation, restriction of differentiation potential, cell signaling and tumorigenesis [19]. After the initial prediction of the existence of few hundred of miRNAs targeting about 30% of human genes [20], current findings suggest that the repertoire of human miRNAs is far more extensive than currently represented by public repositories (< 3.000) [21], including those recently identified as key regulatory molecules of immune function [22, 23] and myocardium remodeling [24].

Further, a sub-class of miRNAs, called circulating miRNAs (cmiRNAs), garnered attention owing to their potential for mediating cell-cell and tissue-tissue cross-talk [25]. These intracellular mediators are produced in various tissues both at baseline and in response to physiological and/or pathological stimuli, and are secreted into the blood where they can be delivered to their respective recipient tissues to modulate gene expression [25]. Finally, must be considered that miRNAs might interact with the chemical epigenetic modifications since the levels of DNA methylation can also influence miRNA levels, while miRNAs can affect translation of enzymes involved in histone modification and DNA methylation [26].

## Physical inactivity and diseases

Epidemiological studies confirm that most people affected by chronic diseases, such as cardiovascular, metabolic and degenerative diseases, have multiple common lifestyle characteristics or behaviours, such as smoking, poor diet, obesity and physical inactivity, that are identified as leading contributors to overall mortality. The potential synergistic effects of multiple lifestyle factors and the risk of chronic conditions and/or health outcomes in adolescence, adulthood and old age are increasingly being recognised [27, 28]. Physical inactivity has been shown to be among the top 10 risk factors for all diseases and is reported to be responsible for 9% of all deaths worldwide with serious health, economic, environmental and social consequences [27]. The most studied of all modern diseases are coronary heart disease (CHD) and metabolic syndrome. Overall, most studies report a negative correlation between levels of PA and the occurrence of these pathologies. Positive effects of PA on the prevention and/or treatment of other pathological conditions such as neurological and cancer diseases are also being reported [29, 30].

Although the beneficial effects of PA are well known, 30% of the world’s population fails to attain the levels of PA recommended for health benefits [31]. During the past years, several studies have shown the potential of aerobic and resistance training either to positively improve specific biomarkers related to different diseases [32] or to reduce the incidence of cardiovascular and metabolic diseases in broad populations of individuals, including women, older individuals, patients with coronary heart diseases [33], diabetes [34, 35] and those with heart failure [33]. The additive effect of aerobic and resistance training has also been demonstrated in individuals affected by conditions such as multiple sclerosis (MS) [36], and chronic pulmonary disease [37]. The American College of Sports Medicine recommends that most adults (18–65 years) engage not only in moderate-intensity aerobic training (≥30 min^.^d^−1^ on ≥5 d^.^wk.^−1^ for a total of ≥150 min^.^wk.^−1^) but also resistance exercise for each of the major muscle groups [38]. Moreover, being physically active can enhance aerobic fitness, strength, power and cognition, overcome fatigue and depression, and improve overall quality of life [39–41].

## Physical activity, epigenetic modifications and diseases: An overview of human studies

Emerging evidence indicates that PA can modulate the epigenetic mechanisms associated with a variety of human diseases [4, 5, 42]. For instance, studies in human monocytic cells [43], granulocytes [44] and peripheral blood mononuclear cells [45] demonstrate that moderate exercise up-regulates the methylation status of apoptosis-associated speck-like protein containing a C-terminal caspase recruitment domain (ASC) [46]. ASC is an important mediator of the cytosol-type inflammatory signaling pathway, and its methylation pattern is associated with the level of pro- and anti-inflammatory cytokines during exercise, regulating the lymphocyte activation and differentiation [47]. These epigenetic mechanisms contribute to lowering the basal level of inflammation, thereby preventing the occurrence of diseases linked to a low-grade chronic inflammation [48]. Moreover, it is recognised that PA counteracts those processes of hypomethylation and hypermethylation associated with neoplastic mutations in the genome [49], thus representing an intervention able to target several genes simultaneously and potentially eliminating any side effects for the patients. Indeed, our group recently demonstrated that 12 weeks of low frequency, moderate intensity power training has the capacity to reduce the global DNA methylation in peripheral mononuclear cells of elderly subjects [50].

Several studies point out that PA acts as a modulator of histone acetylation, particularly H3 and H4, in different tissues [15], promoting chromatin modification that can lead to selective transcription or inhibition of specific genes related to cancer [51], muscle wasting [52] or behavioral [53] diseases.

During the last years, a substantial increase of data suggests that PA may affect the production of miRNAs [54]. In particular, among the over 100 miRNAs which have been found to be modulated in response to exercise, some are involved in specific cancers [55–58], metabolic [59] and cardiovascular diseases [60].

The most significant human studies investigating the relationship between epigenetic modification and PA are summarised in Table 1 and reveal the capacity of PA to preserve and/or recover the “positive” epigenetic markers that are known to be modified in important chronic diseases such as cancer, metabolic, cardiovascular and neurodegenerative diseases. Readers interested in other environmental factors that may have the potential to modulate epigenetic modifications (i.e. tobacco smoke, dietary components, and other exogenous factors) are referred to other thematic reviews [61].

**Table 1:** Main outcomes of human studies on physical activity, epigenetics and diseases

	Type of study	Subjects	PA assessment	Tissue Analysed	Results	References
Cancer disease	OS	45 healthy female (43±7 yrs)	PA questionnaire (for the past years, the past 5 years and over a lifetime)	Breast	No significant correlation between PA and APC or RASSF1A methylation	[62]
	OS	1154 people with colon cancer (30-79 yrs)	PA questionnaire (for the current year, the past 10 and 20 years)	Colon	No significant correlation between PA and number of methylated markers	[69]
	OS	106 people with gastric cancer (59-66 yrs)	PA questionnaire (referent before cancer oneset)	Gastric	Significant correlation between PA and CACNA2D3 methylation.	[64]
	OS	750 people with rectum tumor (30-79 yrs)	PA questionnaire (for the current year, the past 10 and 20 years)	Rectum	No significant between PA and CIMP tumors	[63]
	OS	131 healthy people (45-75 yrs )	PA assessed over 4 days	Leukocytes	No significant correlation between PA and LINE-1 methylation	[65]
	OS	4654 healthy people (55-69 yrs)	PA self-reported (occupational history)	Colorectal	No significant association between PA and CIMP tumors	[66]
	IS	12 female with breast cancer (55-65 yrs)	6 months of 2.5hrs per week of moderate intensity treadmill exercise	Leukocyte and breast	Significant association between PA and DNA methylation in a tumor suppressor gene (L3MBTL1)	[67]
	OS/IS	64 healthy male and female (29±8 yrs)	PA self-reported over 7 days and 12 months	Buccal cells	Significant correlation between PA and DNA methylation of genes associated with carcinogenesis process	[68]
	OS	647 healthy female (35-73 yrs)	PA self-reported at different times in life (childhood, teenage years and past 12 months)	Leukocytes	No significant correlation between PA and LINE-1 DNA methylation	[74]
	IS	12 healthy men (22.3 ± 1.0 yrs)	Acute aerobic exercise ( 10, 2-min bouts of cycle ergometer exercise at a constant sub-maximal workload interspersed with 1-min rest)	Leukocytes	PA alter the expression of 986 genes and 23 miRNAs associated with cancer and cell communication in NK cells	[44]
Metabolic disease	IS	14 young male and female (25±1 yrs)	2 sessions of acute exercise: low-intensity (40% V̇O_2max_); high intensity (80% V̇O_2_max)	Vastus lateralis	Significant hypometilation of PGC-1α, TFAM, PPARD, PDK4	[78]
	IS	15 men T2D FH^+^, 13 men T2D FH^-^ (37.5±5.2 yrs)	6 months aerobic exercise (3hrs per week: 1 session of 1h spinning; 2 sessions of 1h aerobic	Vastus lateralis	Significant correlation between PA and the methylation of markers associated with T2D (RUNX1, MEF2A, THADA, NDUFC2, ADIPOR1, ADIPOR2, BDKRB2)	[84]
	IS	31 men, 15 T2D FH+ and 16 T2D FH- (37.5±5.2 yrs)	6 months aerobic exercise (3hrs per week: 1 session of 1h spinning; 2 sessions of 1h aerobic	Adipose	Significant correlation between PA and the methylation of markers associated with obesity and T2D	[85]
	IS	17 T2D patients (13 female, 5 male) ( 49±5 yrs)	16weeks endurance exercise on cycle ergometer for 1 h/session at 60% V̇o2 peak (3 time per week)	Vastus Lateralis	Significant hypomethylation of the promoter region for NRF1, hypomethylation at a CpG shelf within the intronic region of the gene body of PFKFB3, and hypermethylation of the promoter region of FASN after exercise.	[59]
CDV disease	IS	12 healthy men (21.1±2.7 yrs)	4 weeks of sprint interval training (3 per week, 249 min in total)	Leukocytes	Training induces specific leukocyte DNA methylation across MIR21 and MIR210	[60]
NDG disease	IS	17 SZ male and female (18-50 yrs)	3 moths combined aerobic and strength training (1h 3 time per week. Aerobic: 20min walking at 60% max cardiorespiratory fitness; strength: 30min of 3 sets of 15 reps major muscle group)	PBMCs	Decrease of global histone H4 acetylation after 30, 60 and 90 days of the intervention.	[53]

*CDV* Cardiovascular, *NDG* Neurodegenerative, *CIMP* CpG Island Methylator Phenotype, *FH+* Family History, *FH-* No Family History, *IS* Interventional study, *OS* Observational study, *PA* Physical activity, *T2D* Type 2 Diabetes, *V̇O*_*2*_ *max* Maximal oxygen uptake

Although the intensity of exercise in published studies are not always specifically measured (e.g. the percentage of maximum oxygen uptake), some studies provide useful information about the frequency and duration of specific exercise protocols able to prevent or treat the course of certain diseases through the preservation or the recovery of “positive” epigenetic markers.

### Cancer disease

Aberrant DNA methylation patterns have been extensively described as triggers for carcinogenesis [13]. Cancer is commonly associated with hypermethylation of tumour-suppressor genes. For example, 5–10% of the thousands of promoter CpG islands that never normally contain DNA methylation become abnormally methylated in various cancer genomes [6]. During the last decade, several studies have attempted to elucidate the effects of PA in relation to methylation of cancer-specific loci, either in term of prevention or treatment. In particular, long-term PA has been shown to impact on epigenetic modulation, reducing the risk and mortality in cancer such as breast, colorectal and gastric cancer [62–68], as detailed in the following paragraphs.

The first observational study conducted by Coyle et al. [62] in 106 healthy females investigated the effects of self-reported PA on methylation of the promoters of the tumor suppressor genes adenomatous polyposis coli (APC) and ras association domain family member 1 (RASSF1A), an epigenetic alteration commonly associated with breast cancer risk. Although not statistically significant, this study found that PA could inversely correlate with the degree of promoter methylation of APC but not RASSF1A in nonmalignant breast tissue. In the same year, Slattery et al. [69] failed to find any significant association between self-reported PA and the methylation levels of five CpG island markers (amyloid beta precursor protein-binding family A member 1, MINT1; amyloid beta precursor protein-binding family A member 2, MINT2; amyloid beta precursor protein-binding family A member 31, MINT31; cyclin-dependent kinase inhibitor 2A, p16^INK4A^; human mutL homolog 1, hMLH1) in 1154 patients with colon cancer. In a subsequent study, the same authors reported no relationship between the five CpG island markers and different lifestyle factors (i.e. nutrients, dietary fiber, body mass index, and long-term PA) in 750 population-based cases of rectal cancer, irrespective of gender [63]. In contrast, Yuasa et al. [64] studied tumors from 106 patients with gastric cancer and investigated the association between PA level, evaluated by self-administrated questionnaire, and the methylation status of several cancer-related genes, such as the homeobox transcription factor (CDX2), the bone morphogenetic protein 2 (BMP2), the GATA binding protein 5 (GATA5), the p16, the calcium voltage-gated channel auxiliary subunit alpha2delta 3 (CACNA2D3) and the estrogen receptor (ER). These authors found that the methylation of CACNA2D3, correlated to gastric cancer [70], was higher in the sedentary carcinoma patients (45.5% of patients) than in those with at least 1 h of exercise per week before cancer onset (23.7% of patients) making this the first positive and specific epigenetic study related to the impact of PA on tumorigenesis. Subsequently, Zhang and colleagues were the first to investigate the correlation between PA and cancer risk through a genome wide analysis of DNA methylation [65]. It is known that global DNA hypomethylation increases genome instability by activating repetitive sequences (e.g. long interspersed nuclear elements, LINEs), which are normally highly methylated [71]. Indeed, a genome wide reduction in DNA methylation is observed in most malignant cells and it is associated with increased cancer risk [72]. In particular, Zhang et al. [65] measured in 161 healthy adult subjects the level of PA assessed by accelerometry over four days and the global methylation in LINE-1 retrotransposons, a marker shown to correlate with global methylation [73]. These authors found global DNA methylation levels to be higher among individuals with 26–30 min/day of PA compared to those with <10 min/day. However, following multivariate adjustment for age, gender, smoking status, ethnicity and body mass index (BMI), this association was no longer statistically significant. The risk of developing colorectal cancer (CRC) in about 4654 patients was assessed by Hughes et al. [66] using the Weisenberg panel of genes to define the CpG Island Methylator Phenotype (CIMP) tumors (calcium voltage-gated channel subunit alpha1 G, CACNA1G; insulin like growth factor 2, IGF2; neurogenin 1, NEUROG1; runt related transcription factor 3, RUNX3; suppressor of cytokine signaling 1, SOCS1) and examined the associations between BMI, waist circumference and self-reported PA level and CRC risk. These authors found no association between the levels of PA (occupational and non-occupational) and the methylation level of CIMP tumors.

The first randomised clinical trial to investigate the effect of exercise on DNA methylation was published by Zeng et al. [67]. These authors investigated the effects of a 6-month clinical exercise intervention (150 min/week of supervised moderate-intensity aerobic exercise on a treadmill) on the methylation profile of 43 genes in blood from a restricted number of sedentary breast cancer patients (*n* = 12; postmenopausal women) using methylation microarrays. Using this approach, these authors found that exercise significantly decreased by 3.6% the methylation level of the L3MBTL1 gene encoding for the lethal (3) malignant brain tumor-like protein 1. Further analysis demonstrated that the changes in L3MBTL1 methylation were related to gene expression in tumor tissues samples from 348 breast cancer patients, and the expression was associated with patient survival. These encouraging data suggest that exercise may lower DNA methylation in certain tumor suppressor genes, thereby inhibiting tumor progression with potential effects on cancer survival. Similarly, encouraging findings were published by Bryan and colleagues [68] who selected 45 CpG sites potentially associated with breast cancer and investigated the association between methylation level and changes in PA and objectively measured cardiovascular fitness. Specifically, 64 healthy participants (82% female), physically inactive at the time of recruitment, were randomized into either a 12-month controlled trial testing an exercise promotion intervention (treadmill exercise, from 50 to 85% maximum heart rate, approximately 30–50 min, 3–5 days per week for 36 weeks) (*n* = 37) or a health-and-wellness contact control condition (*n* = 27). Despite the small numbers, significant associations were reported between buccal cell DNA methylation and both objectively measured cardiovascular fitness and PA at baseline while only the change in PA duration was associated with a decrease in methylation after 12 months. These results lead to the speculation that higher levels of PA may be needed to achieve a “healthier” methylation profile at CpG islands of genes associated with carcinogenic processes. More recently, White and colleagues [74] investigated the association between self-reported PA at different life stages (e.g. childhood, adolescent, and previous 12 months) and LINE-1 methylation in a sample of 600 non-Hispanic white women with a family history of breast cancer. PA medians in hours per week were 12.5 for past 12 months, 5.9 for adolescent, and 9.8 for childhood. The women that reported a PA status above the median for all three periods, showed a significant higher LINE-1 methylation than those below the median, while no statistical differences were found in women who were at or above the median, for 1 or 2 of the time periods.

So far, no human studies show direct correlation between PA, miRNAs and cancer diseases. Nevertheless, it has been shown that brief, acute exercise (ten 2-min bouts of cycle ergometer exercise at a constant sub-maximal workload interspersed with 1-min rest) significantly alters the expression of 986 genes and 23 miRNAs predominantly associated with cancer and cell communication in natural killers (NK) cells of 12 healthy men [44]. Moreover, in 2016, Isanejad et al. [75] demonstrated in a typical animal model of breast cancer that the reduction of tumor growth determined by 5 weeks of interval training intervention in combination with the hormone therapy was associated with the decreased expression of miRNA-21 and the increased expression of miRNA-206 and miRNA let-7. Therefore, it seems that the modulation of pathways related to miRNAs expression might be involved in anti-tumorigenic effects of exercise.

**Fig. 1:**
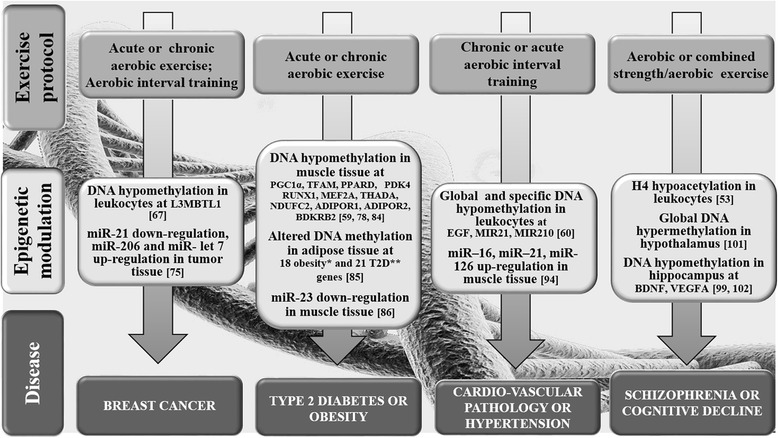
Small-scale intervention protocols from human and animal studies focusing on the exercise-related epigenetic modulations in human diseases and/or in specific disease candidate genes. * Exercise-induced hypermethylation (*ADAMT59, CPEB4, GRB14, ITPR2, LY86, LYPLAL1, MAP2K5, MSRA, MTIF3, MRXN3, PRKD1, SOCCAG8, STAB1, TBX15, TMEM160, ZNF608)* or hypomethylation (*GPRC58, TUB)* in obesity candidate genes [85]. **Exercise-induced hypermethylation (*ADAMT59, ADCY5, ARAP1, BCL11A, CDKAL1, CDKN2A, DGKB, DUSP8, FTO, HHEX, HMGA2, IGF2BP2, JAZF1, KCNQ1, PRC1, PROX1, PTPRD, TCF7L2, THADA, WFS1, ZBED3)* or hypomethylation (*KCNQ1, TCF7L2)* in T2D candidate genes [85]

Most of the borderline/contradictory findings summarized here have emerged from small studies requiring replication in larger clinical trials. Despite the limitations of the current literature and the need for replication, these studies, in general, suggest a long physically active lifestyle could be very important in inhibiting tumor proto-oncogene and decreasing cancer risk (Fig. 1).

### Metabolic disease

There is also emerging evidence that epigenetic factors may, at least in part, explain not only the beneficial effects of exercise on the prevention and treatment of type 2 diabetic patients (T2D) and other metabolic disorders [76], but even the beneficial effects on metabolic outcomes of offsprings (e.g. glucose tollerance and glucose clearance) through the maternal transmission of epigenetic modifications of genes involved in important metabolic pathways [77].

Specifically, exercise has been shown to cause hypomethylation of metabolic genes promoters such as peroxisome proliferator-activated receptor gamma coactivator 1-alpha (PGC-1α), mitochondrial transcription factor A (TFAM), peroxisome proliferator-activated receptor delta (PPARD), pyruvate dehydrogenase lipoamide kinase isozyme 4 (PDK4), citrate synthase, and myocyte enhancer factor 2A (MEF2A), many of which are hypermethylated in T2D patients [78]. Aerobic exercise has also been shown to reduce the expression of various types of miRNAs in human skeletal muscle, 22% of which target genes that regulate transcription and 16% target genes involved in muscle metabolism, especially in oxidative phosphorylation [79]. Since 2000, genome-wide association studies focusing the attention on the identification of T2D genetic variants, considering lifestyle intervention as a putative key factor for the prevention and the development of the disease [80]. Several studies have highlighted the role of epigenetic changes in target tissues from patients affected by T2D. Indeed, DNA methylation of the PPARG coactivator 1 alpha (PPARGC1A), a gene that coordinates the expression of several proteins involved in mitochondrial oxidative metabolism in multiple tissues, is elevated in both pancreatic islets [81] and skeletal muscle [82] of patients with T2D, and negatively correlates with glucose-stimulated insulin secretion in human pancreatic islets. In addition, a previous study of human islet-derived precursor cells has reported that the insulin gene displays hyperacetylation of H4 and hypermethylation of H3 at lysine 4 [83]. Barres et al. [78] focused on the possible link between epigenetic modification, T2D and the effects of PA. They assumed that a specific exercise regime, that included two acute exercise trials at 40% (low-intensity) and 80% (high-intensity) of maximal oxygen uptake on separate occasions, would improve metabolic efficiency, oxidative capacity, and contractile activity in skeletal muscle by altering the DNA methylation levels of genes that were differently methylated in T2D. Results obtained from 14 muscle biopsies of healthy sedentary subjects demonstrated that exercise markedly reduced promoter methylation of PGC1α, TFAM, PPARD, and PDK4. This is the first evidence that gene-specific DNA hypomethylation could be induced by acute exercise in human skeletal muscle. Similar results were reported by Nitert and colleagues [84], as they identified 65 genes exhibiting differential DNA methylation in muscle of male subjects (*n* = 15) with at least one first-degree relative with T2D (FH^+^) compared with same gender subjects (*n* = 13) without a family history for this disease (FH^−^) at baseline. Of these, 21 T2D candidate genes showed differential DNA methylation in FH^+^ compared with FH^−^men. They also found that a 6-month exercise intervention (3 h per week: 1 session of 1 h spinning and 2 sessions of 1 h aerobic) decreased DNA methylation of eight T2D candidate genes (runt related transcription factor 1, RUNX1; myocyte enhancer factor 2A, MEF2A; THADA armadillo repeat containing, THADA; NADH:ubiquinone oxidoreductase subunit C2, NDUFC2; adiponectin receptor 1, ADIPOR1; adiponectin receptor 2, ADIPOR2; bradykinin receptor B2, BDKRB2; RNA binding motif single stranded interacting protein 1, RBMS1). Subsequently, Ronn and Ling [85] performed a study on subcutaneous adipose tissue biopsies taken at baseline from individuals with or without family history of T2D. Both groups were combined to evaluate the impact of exercise on the global human methylome in adipose tissue, calculating the average level of DNA methylation in groups based on the functional genome distribution, the CpG content or the neighbourhood context. They showed a relevant increase of global DNA methylation in adipose tissue after 6-months of exercise, with 17,975 individual CpG sites exhibiting differential DNA methylation in adipose tissue, corresponding to 7663 unique genes throughout the genome. Notably in this study, 18 obesity and 21 T2D candidate genes had CpG sites with differences in DNA methylation in response to exercise.

Recently, Rowlands et al. [59] constructed an integrated epigenomic-transcriptome networks from multi-omic microarray analysis of skeletal tissue from 17 adults people with T2D in response to 16 wk. of endurance training. Their data revealed a link between the endurance training and several epigenetic modifications related to the molecular reprogramming of lipid and glucose processing pathways. In particular, the authors found hypomethylation of the promoter region of glucose transporter type 4 (GLUT4) and NRF1 (Nuclear Respiratory Factor 1), a key transcription factor of several genes regulating cell growth, mitochondrial respiratory proteins, mitochondrial DNA transcription and replication, as well as a hypomethylation at a CpG within the intronic region of the gene body of 6-phosphofructo-2-kinase/fructose-2,6-biphosphatase isoform 3 (PFKFB3) and within an exon region of glycogen synthase kinase 3α (GSKA). These enzymes determine the glycolytic rate via the biosynthesis and degradation of fructose 2,6-bisphosphate and the rate of glycogen synthesis, respectively, and therefore contribute toward muscle capacity for glucose uptake, storage, and utilization. Further, they discovered a hypermethylation of the promoter region of FASN (Fatty acid synthase), which may contribute to the observed decrease in intramyocellular lipid by lower FASN activity leading to reduced fatty-acid synthesis and lipid accumulation. Lastly, the authors demonstrated that exercise training reduces miR-29a expression, suggesting the epigenome-transcriptome connection in lipid metabolism, cellular growth, apoptosis and GLUT4 translocation [59].

A further confirmation on the role of mirRNAs in the regulation of mitochondrial biogenesis, glucose and fatty acid metabolism, derives from the study coducted by Safdar et al. [86] in mice, where the down-regulation of miR-23 is associated with a significant increase in PGC-1 αmRNA expression and protein content in quadriceps of C57Bl/6 J male animals three hours following an acute bout of endurance exercise.

In summary, it appears that aerobic exercise training alters in a dose-dependent manner not only the levels of global methylation but also the level of gene-specific promoter methylation, improving the expression of genes involved in metabolic diseases, especially those related to T2D (Fig. 1).

### Cardiovascular disease

It is well established that PA has a positive impact on a wide range of biological functions, counteracting those molecular mechanisms able to alter gene expression in cardiovascular [33, 87]. For instance, some heart diseases (e.g. human dilated cardiomyopathy) are characterized by a deregulated expression of both coding and non-coding DNA sequences directly affecting heart failure development and progression [87]. In particular, Movassagh and colleagues [88] demonstrated that cardiomyopathies show different global DNA methylation profiles with respect to a normal control, and that some of these profiles can be reversed through a specific modulation of miRNA.

At present, there is only indirect evidence connecting epigenetic changes and cardiovascular adaptations to exercise and PA in humans with particular reference to heart and vessels. For example, it is thought that the deregulation of specific epigenetic mechanisms depends upon the ratio of histone acetyltransferases /histone deacetylase ((HAT/HDAC) or by their respective function, leading to modified expression of matrix metalloproteinases (MMPs), which are related to pathological alterations of vascular walls [89], altered proliferation of endothelium myocytes in heart and vessels [90], or lethal cardiomyopathy [91]. Therefore, regular physical exercise may have a protective role against MMP-related cardiovascular alteration by restoring HAT and HDAC activity to normal [92]. In addition, exercise training causes a non-pathological increase of the myocardial mass, resulting in cardiac hypertrophy and neo-angiogenesis [93]. This process would appear to be orchestrated by numerous miRNAs, which in turn regulate their target mRNAs and, thus, provoke physiological cardiac hypertrophy through different signaling pathways [24].

To date, a study conducted by Denham et al. [60] represents the only interventional study that correlates epigenetic changes induced by exercise and cardiovascular system in human subjects. Specifically, DNA methylation and transcriptome analysis was performed on leukocytes obtained from 12 healthy young men at rest and after 4 weeks of sprint interval training, a form of exercise training that rapidly improves vascular functioning. The authors demonstrate global and specific changes in DNA methylation that were linked with changes in the expression of miRNA and protein-coding genes associated with cardiovascular physiology, like mir-21, mir-210 and epidermal growth factor. Data obtained from animal studies seem to confirm that aerobic exercise can prevent and cure hypertension by miRNAs modulation. Indeed, after 10 weeks of training (60 min of swimming, 5 times/week) the levels of miRNA-16, miRNA-21, and miRNA-126, were restored, preventing the microvascular abnormalities in hypertension through the perfect balance between angiogenic and apoptotic factors [94].

In summary, exclusive sprint interval training (maximal aerobic exercise) seems to be associated with epigenetic modifications known to positively influence cardiovascular adaptation.

### Neurodegenerative disease

Emerging experimental and clinical evidences suggest the imbalance of epigenetic machinery on neurological disease. Epigenetic modifications of specific genes (i.e. synuclein alpha, SNCA; leucine rich repeat kinase 2, LRRK2; parkin RBR E3 ubiquitin protein ligase, PARK2; parkinson disease 16, PARK 16/Iq32; glycoprotein nmb, GPNMB and histone deacetylases, (HDAC) seem to be implicated in neurological disorders (e.g. epilepsy, schizophrenia, Alzheimer and Parkinson diseases) [95–98] and although most of the evidence in this field derive from animal models, numerous studies in the last few years have shown that the central nervous system plasticity is subjected to epigenetic regulations induced by exercise.

To date, the work conducted by Lavratti et al. [53] represents the first human study that demonstrates a relation between exercise training and levels of global histone acetylation in people affected by neurodegenerative diseases. In particular, they found that 90 days of combined exercise program (1 h, 3 times/week, aerobic and strength training) was able to induce a significant histone H4 hypoacetylation status in PBMCs of schizophrenia patients, indicating a reduced transcriptional activity and gene expression. Although it is impossible to establish the clinical relevance of this data, the authors tentatively suggest that exercise could transcriptionally silence genes that exert a pivotal role in the physiology and progression of schizophrenia through epigenome modulation [53]. It has been well demonstrated in animal model that the promoter IV of brain-derived neurotrophic factor (BDNF), a neurotrophin highly expressed in hippocampus and important for the neuronal development, undergoes reduced CpG methylation in the rat following regular engagement in physical exercise [99] and that free-wheel running (from 1.6 to 7 km/day) may enhance histone H3 phospho-acetylation and c-Fos induction in dentate granule neurons [100]. Recently, Kashimoto et al. [101] reported that exercise increases the global DNA methylation profile in the hypothalamus of Wistar rats submitted to swimming and may modulate epigenetic responses evoked in the hippocampus, cortex, and hypothalamus by repeated restraint stress. Similarly, Sølvsten et al. [102] demonstrated that voluntary exercise in rat induces a DNA hypomethylation at specific CpG site located within a VegfA promoter Sp1/Sp3 transcription factor recognition element. Moreover, they found a significant reduction of DNA methyltransferase (Dnmt3b) mRNA in the hippocampus of exercised rats, pointing to an eventual genome-wide DNA hypomethylation in brain in response to exercise.

Despite the somewhat encouraging results from human and animal studies, which substantiate the involvement of epigenetic mechanisms as mediators of the beneficial effects of exercise, it remains difficult to extrapolate useful information to set up an exercise intervention to improve the epigenetic profile of individuals at risk of, or affected by neurodegenerative diseases (Fig. 1). Therefore, further studies are needed to delineate the mechanisms behind the functional impact of physical exercise in mediating health benefits to the brain tissues.

## Conclusions

Numerous studies have attempted to investigate whether modification of lifestyle factors, especially increasing PA levels, can influence the epigenetic patterns involved in human cancer, metabolic, cardiovascular and neurodegenerative diseases. However, in most of the observational studies, the information about PA is self-reported via questionnaires during the study period or during restricted periods (e.g. such as before the disease onset, the past week, the past year, the past 5 years) and without sufficient information about the exercise type and/or exercise dose. It is now acknowledged that self-reported PA assessed by questionnaire is not sufficiently accurate for individual assessment [103] and this is most likely the reason why many studies fail to demonstrate a strong association between PA and epigenetic markers: the possibility of recall errors from the participants may have generated an important bias, making it difficult to extrapolate and use the findings for interventional and prescriptive purposes. Moreover, most of the human studies do not control for other environmental or perinatal factors that may impact the epigenetic mechanisms or their interconnection.

The encouraging results presented throughout this review call for large-scale, collaborative efforts involving large, well-phenotyped cohorts [104]. A better approach to address this issue would be to set up international consortia with a particular focus on interventions with proven efficacy where the PA of participants will be objectively assessed by accelerometry and the exercise intervention defined in terms of intensity (e.g. percent of maximum oxygen uptake and/or maximum heart rate), frequency, duration, and objectively monitored. With this in mind, it is worth mentioning recent initiatives calling for international collaborative efforts to the investigation of genomic and other “omic” markers of sports and exercise performance, such as the Athlome Project Consortium [105]. It currently consists of 15 participating centres worldwide focusing on identifying, among others, the epigenetic alternations influencing athletic performance, such as the Gene SMART (*Skeletal Muscle Adaptive Response to Training*) project, related to the genomics, transcriptomics and proteomics modifications that predict the skeletal muscle response to high-intensity interval training, the NTR (Netherlands Twin Register), focusing on the interplay between genetic and environmental factors shaping individual differences in sports participation and performance, or the LCR-HCR study (based on the *Low Capacity Rats-High Capacity Rats* model), aiming to explore the correlation between intrinsic endurance exercise capacity and risk to develop diseases like metabolic syndrome, premature aging, obesity, and Alzheimer [106].

Moreover, we have to consider that epigenetic patterns are known to change between tissues, and even in a cell-specific manner within some tissues. Indeed, the specific impact of PA on epigenetic modifications of different human tissues is unknown. Most epidemiological or interventional studies to date have been conducted on tissues not always involved in the examined diseases. Differences but also similarities between tissues in terms of exercise-induced epigenetic changes should also be assessed.

In conclusion, PA promises to be an important tool to be used alone or in combination with traditional therapies to improve the efficacy of strategies for disease prevention and treatment based on epigenetic modification. In this context, exercise remains an essential factor promoting important biological adaptations with profound implications for public health. Future collaborative studies may identify epigenetic markers with translational significance in identifying individuals for whom a personalized exercise regime could significantly alter the epigenomic signature and thus the risk of disease development or progression.
